# Antimicrobial Prophylaxis in Robot-Assisted Laparoscopic Radical Prostatectomy: A Systematic Review

**DOI:** 10.3390/antibiotics12121744

**Published:** 2023-12-16

**Authors:** Eva Falkensammer, Ece Erenler, Truls E. Bjerklund Johansen, Lazaros Tzelves, Laila Schneidewind, Yuhong Yuan, Tommaso Cai, Bela Koves, Zafer Tandogdu

**Affiliations:** 1 Department of Urology, Klinikum Wels-Grieskirchen, 4600 Wels, Austria; 2 Department of Pediatric Surgery, University Hospital Salzburg, 5020 Salzburg, Austria; 3 School of Medicine, Koc University, Istanbul 34450, Turkey; eerenler19@ku.edu.tr; 4 Department of Urology, Oslo University Hospital, 0424 Oslo, Norway; t.e.b.johansen@medisin.uio.no; 5 Institute of Clinical Medicine, University of Aarhus, 8000 Aarhus, Denmark; 6 Department of Urology, National and Kapodistrian University of Athens, Sismanogleio General Hospital, 11527 Athens, Greece; 7 Department of Urology, University Greifswald, 17487 Greifswald, Germany; laila.schneidewind@uni-greifswald.de; 8 Department of Medicine, London Health Science Centre, London, ON N6A 5W9, Canada; Yuhong.Yuan@lhsc.on.ca; 9 Department of Medicine, Health Sciences Centre, McMaster University, Hamilton, ON N6A 5W9, Canada; 10 Department of Urology, Santa Chiara Hospital, 38123 Trento, Italy; ktommy@libero.it; 11 Institute of Clinical Medicine, University of Oslo, 0424 Oslo, Norway; 12 Department of Urology, South-Pest Teaching Hospital, 1097 Budapest, Hungary; 13 Department of Urology, University College London Hospitals, London NW1 2BU, UK; zafer.tandogdu@nhs.net

**Keywords:** antimicrobial prophylaxis/antibiotic prophylaxis (AP), postoperative infection, prostate cancer (PCa), robot-assisted laparoscopic radical prostatectomy (RALP), sepsis, surgical site infection (SSI), urinary tract infection (UTI)

## Abstract

It remains unclear whether antibiotic prophylaxis (AP) should be recommended or discouraged in robot-assisted laparoscopic radical prostatectomy (RALP) for prostate cancer (PCa). The development of microbial resistance and side effects are risks of antibiotic use. This systematic review (SR) investigates the evidence base for AP in RALP. A systematic literature search was conducted until 12 January 2023, using Embase, MEDLINE, Cochrane CENTRAL, Cochrane CDSR (via Ovid) and CINAHL for studies reporting the effect of AP on postoperative infectious complications in RALP. Of 436 screened publications, 8 studies comprising 6378 RALP procedures met the inclusion criteria. There was no evidence of a difference in the rate and severity of infective complications within 30 days after RALP surgery between different AP protocols. No studies omitted AP. For patients who received AP, the overall occurrence of postoperative infectious complications varied between 0.6% and 6.6%. The reported urinary tract infection (UTI) rates varied from 0.16% (4/2500) to 8.9% (15/169). Wound infections were reported in 0.46% (4/865) to 1.12% (1/89). Sepsis/bacteraemia and hyperpyrexia were registered in 0.1% (1/1084) and 1.6% (5/317), respectively. Infected lymphoceles (iLC) rates were 0.9% (3 of 317) in a RALP cohort that included 88.6% pelvic lymph node dissections (PLND), and 3% (26 of 865) in a RALP cohort where all patients underwent PLND. Our findings underscore that AP is being administered in RALP procedures without scientifically proven evidence. Prospective studies that apply consistent and uniform criteria for measuring infectious complications and antibiotic-related side effects are needed to ensure the comparability of results and guidance on AP in RALP.

## 1. Introduction

Robot-assisted laparoscopic radical prostatectomy (RALP) is the preferred treatment option for localized prostate cancer (PCa) [1]. Currently, antibiotic prophylaxis (AP) is broadly administered in RALP to prevent post-surgical infections. Extensive prophylactic administration drives antimicrobial resistance (AMR), which poses a significant threat to global health and patient safety [2]. The role and duration of AP for open, laparoscopic and robot-assisted prostatectomy is not well documented. A SR of randomized controlled trials addressing the periinterventional antibiotic prophylaxis in urologic open and laparoscopic urologic surgeries was unable to identify relevant studies [3]. The Global Prevalence Study on Infections in Urology (GPIU) revealed that misuse of antibiotics for prophylaxis is the case in 84% of urological procedures [4]. According to the GPIU, nearly half of hospitalized urological patients, 3898/8178 (47.7%), across 60 countries between 2005 and 2010 received antibiotics for periprocedural prophylaxis [4]. The overuse of AP is echoed in other surgical specialties and has been identified by the WHO as a key area of intervention to tackle AMR [2]. 

The current practice of administering prophylactic antibiotics in RALP is based on the 1999 Guideline of Surgical Site Infection developed by the Centers for Disease Control and Prevention (CDC) [5]. According to the Altemeier classification, RALP falls under the category of Class II/ Clean-Contaminated surgery. However, the presence of preoperative bacteriuria upgrades the procedure to the contaminated category. In such cases, therapeutic antibiotics based on the antimicrobial susceptibility of the urinary culture results are warranted, rather than single or extended prophylactic antibiotic courses [5]. The latest update on perioperative antibiotic prophylaxis by the EAU guideline panel states that the scientific evidence regarding the use of antibiotic prophylaxis before prostatectomy (PE) was insufficient to provide a definitive recommendation on antibiotic prophylaxis protocols for RALPs [6].

This systematic review aims to investigate the evidence base for AP in RALP. Our primary objective was to identify papers that demonstrate that AP reduces the rate and severity of postoperative infections. Our secondary aim was to identify papers that present the rate and severity of infectious complication rates with and without AP, respectively. Our tertiary aim was to analyze patient demographics and other factors which possibly increase the risk of infection and adverse effects of AP. 

## 2. Methods

*Definitions Used*

Antibiotic prophylaxis was defined as the periprocedural systemic administration of an antimicrobial agent in RALP surgery. A single-dose AP was defined as the administration of a single dose prophylactic antimicrobial agent within 120 min before, at the start or within 30 min after the surgical incision. Short-term AP was defined as continuing the antibiotic regimen for a maximum of 24 h and then discontinuing it. Long-term AP was characterized by the use of antibiotics for a duration exceeding 24 h after the RALP intervention. AP was regarded as the intervention in all included studies.

*Study Variables*

The primary outcome variable was the difference in the rate and severity of infective complications within 30 days after RALP surgery with and without AP prophylaxis. Secondary outcomes were the rate and severity of infective complications within 90 days after RALP surgery with or without AP. Tertiary outcomes were rates of harmful effects of AP, such as *Clostridioides difficile* infections, yeast infections, allergic reactions, rash, diarrhoea and nausea.

Comparative interventional studies enabled comparison of the rate and severity of infections and AP adverse effects between different AP protocols and surgical approaches. Observational case series captured the rate and severity of infections and AP adverse effects in single cohorts without comparators.

*Literature Search*

This systematic review was conducted in accordance with the Cochrane Handbook of Systematic Reviews [7] and the Preferred Reporting Items for Systematic Review and Meta-analysis (PRISMA) reporting guidelines [8]. The protocol was registered with PROSPERO (registration number: CRD42023385517) [9].

### 2.1. Search Strategy

Searches were conducted in five electronic databases without language restriction: MEDLINE (via Ovid; from 1946 to 12 January 2023), EMBASE (via Ovid; from 1974 to 12 January 2023), Cochrane CENTRAL (Cochrane Central Register of Controlled Trials) and Cochrane CDSR (Cochrane Database of Systematic Reviews) (via Ovid; from 2005 to 12 January 2023) and CINAHL (via EBSCO; 1937 to 12 January 2023). The search terms utilized in each database are presented in the supplementary (Supplementary Materials). The reference lists of included studies were also screened for relevant studies.

### 2.2. Study Eligibility

Randomized control trials, non-randomized controlled trials, cohort studies (prospective or retrospective), cross-sectional studies, case-controlled studies and single-arm studies (with at least 10 patients) were considered eligible for this systematic review. Previous systematic reviews, case reports, expert opinions, comments, editorials and conference abstracts were excluded. 

Only studies that documented occurrences of infective complications within at least 30 days after RALP and that explicitly disclosed the utilization of AP were included in the systematic review. Specifics concerning the precise antibiotic agent employed or the schedule of antibiotic administration were not mandated as a prerequisite for inclusion. Nevertheless, it was obligatory for the studies to stipulate whether any prophylactic antibiotics had been administered. In instances where pertinent AP data were absent, these studies were excluded from analysis, as the ability to establish a correlation between infection rates and AP protocols remained hampered.

### 2.3. Selection of Studies

Two authors (E.F. and E.E.) independently screened titles and abstracts to determine which studies should be assessed further in full text. All selected articles were reviewed independently on inclusion criteria and study design. In case of discordance, a third reviewer was consulted (L.T.). Discrepancies were resolved through consensus or consultation with a third review author (L.T.). Papers that were not included in the systematic review were, however, subjected to a general review.

### 2.4. Data Extraction

Data extraction was performed independently by two authors (E.F. and E.E.), using a pre-set Excel sheet containing baseline characteristics (year of publication, country, period of study, sample size, number of surgeons, preoperative urinary culture, operation time, pelvic lymph node dissection, patient demographics, length of stay, indwelling catheter time, wound drainage, performance of cystogram, follow-up period, mode of follow-up, AP schedule), and primary and secondary outcomes. We also registered how infections were measured and defined. 

### 2.5. Risk of Bias Assessment

Two authors (E.F. and L.T.) independently assessed risk of bias among studies using the Cochrane ROBINS-I (Risk Of Bias in Non-randomised Studies of Interventions) [10] tool for comparative studies and the JBI Critical Appraisal Tool [11] for Case Series. Minor disagreements regarding bias due to confounding and bias in measurement of outcomes were resolved by consulting a third author (L.S.).

### 2.6. Assessment of Study Heterogeneity and Data Synthesis

Due to the different designs of included studies and endpoints, the results of this systematic review are reported in a descriptive manner. 

## 3. Results

*Evidence Base*

**Figure 1:**
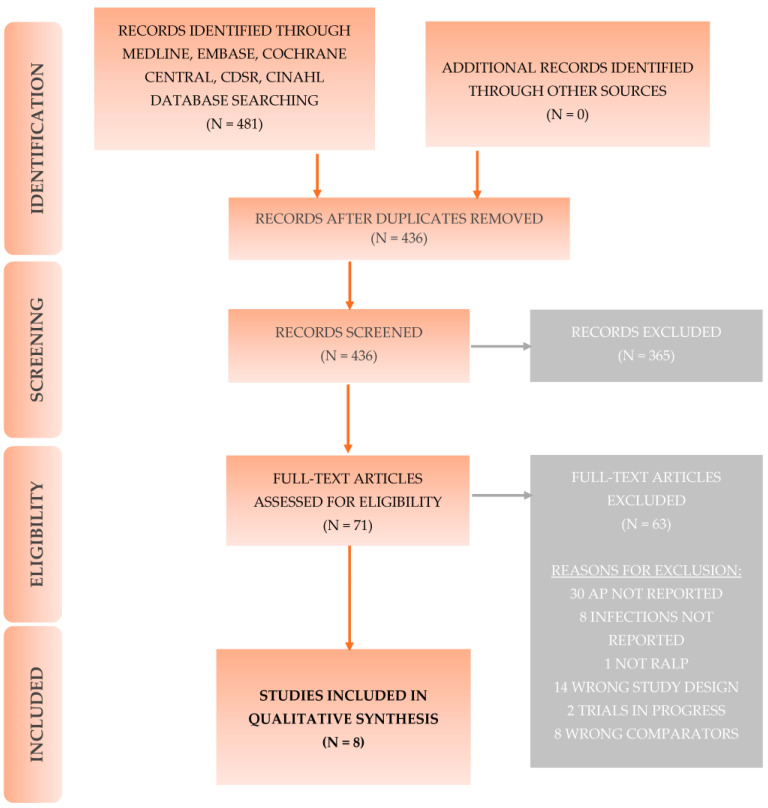
Prisma flow diagram: Screening process of the literature [8].

The screening process of identified studies is depicted in the PRISMA Flow diagram (Figure 1). After reviewing 436 studies and eliminating duplicates, 8 studies met the criteria for inclusion in the systematic review. 

### 3.1. Study Design

No randomized controlled trials (RCTs) directly compared the infectious outcomes of different AP protocols, and no studies compared AP administration with a placebo.

Among the included studies, two comparative cohort studies examined infectious complications between RALP and open PE [12,13], and two other comparative cohort studies compared single-dose AP with long-term AP [14,15]. 

Four case series were identified, with Ferrari et al. employing a long-term AP approach [16], Ahmed et al. using short-term AP [17], and two case series utilizing single-dose AP [18,19]. All studies were conducted retrospectively, except for the study by Ferrari et al., which was prospective [16].

#### 3.1.1. Comparative Cohort Studies on RALP vs. Open PE

Tollefson et al. reported significantly lower incisional SSI rates in RALP compared to open PE (0.6% vs. 4.5%, *p* < 0.001), but rates of UTI (1.6% vs. 1.2%, *p* = 0.28) and sepsis/bacteremia (0.1% vs. 0.1%, *p* = 1) did not differ statistically by surgical approach [8]. Shigemura et al. found a lower overall occurrence of postoperative infections in RALP compared to open PE (1.12% vs. 4.77%, *p* = 0.08), without statistically significant difference [13].

#### 3.1.2. Comparative Cohort Studies on Long-Term AP vs. Single-Dose AP

Haifler et al. observed similar rates of CAUTIs before and after transitioning from long-term to single-dose AP (8.3% vs. 8.9%, *p* = 0.89) [14]. Hartung et al. reported no statistically significant difference in wound infection and UTI rates between long-term and short-term AP (9.72% vs 11.88%, *p* = 0.5) in pooled RALP and open PE cohorts. [15]. Hartung was contacted to inquire about separate infection rates for the robotic and open surgical approaches, but it was confirmed that the infection rates were only calculated within the pooled RALP and open PE cohorts.

#### 3.1.3. Case Series with Long-Term AP

Ferrari et al. observed infectious complications in 6.6% of all patients, with UTIs being the most common at 2.5% and wound infections the least common at 0.6% [16]. The study also documented infected lymphoceles (iLC) in 0.9% of the pooled RALP cohort of whom 88.6% had PLND. The overall complications, including non-infectious ones, were collected up to 90 days after the intervention; 5.2% of overall complications occurred 30 days after the procedure.

#### 3.1.4. Case Series with Short-Term AP

Ahmed et al. reported a lower overall infection rate of 0.6%, including 0.3% of *C. difficile* enterocolitis, 0.2% of UTI cases, and 0.1% of upper respiratory infections [17].

#### 3.1.5. Case Series with Single-Dose AP

Coelho et al. observed lower rates of wound infections (0.56%) and UTIs (0.16%) [18]. Hamada et al. found a 0.46% frequency of wound infection and 3% rate of iLC; of these, 85% had a monomicrobial positive fluid culture caused by Gram+ cocci. The gastrointestinal tract and skin flora were considered as the main sources of infection for iLC [19].

### 3.2. Outcomes

#### 3.2.1. Primary Outcome

There was no evidence of a difference in the rate and severity of infective complications within 30 days after RALP surgery between different AP protocols. 

#### 3.2.2. Secondary Outcomes

We did not identify any control arms or cohorts that reported the rate of infective complications in patients undergoing RALP who did not receive AP. No study omitting AP was identified. For patients who did receive AP, the overall occurrence of postoperative infectious complications varied between 0.6% and 6.6% [16,17]. Reported UTI rates in RALP ranged from 0.16% to 8.9% [14,18]. The incidence of wound infections was reported to be 0.46% and 1.12% in two different studies [13,19]. Sepsis/bacteremia and hyperpyrexia were observed in 0.1% [12] and 1.6% [16] of cases, respectively. The results, including identified AP schedules and reported infectious complication rates, are summarized in Table 1. Cephalosporins were the most commonly administered preoperative AP agents.

#### 3.2.3. Tertiary Outcomes

The rates of iLC were 0.9% in a RALP cohort including 88.6% of cases with PLND and 3% in a RALP cohort where all patients underwent PLND [16,19]. 

Significant heterogeneity in the classification of infectious complications was observed among the studies (Table 2). The table also indicates which studies consistently employed the official CDC definitions for categorizing infectious complications and the duration of follow-up periods.

Most studies, with the exception of Hamada et al. and Ferrari et al., which reviewed records up to day 90, applied 30 days as the follow-up period. Coelho et al. was the sole identified study with a systematic follow-up of patients after hospital discharge, while the remaining studies reviewed in-hospital records or did not specify the follow-up procedure [18]. 

*C. difficile* colitis was mainly measured as a side effect, with Ahmed et al. reporting 0.3% incidence [17]. Haifler et al.’s retrospective study mentioned no cases of *C. difficile* colitis and the other studies did not report on this outcome [14]. Other potential side effects were not mentioned by the eligible studies.

### 3.3. Risk of Bias Assessment 

The risk of bias assessment for the four included comparative non-randomized studies of interventions (NRSI) revealed some concerns, leading to a high overall risk of bias affecting the quality of evidence. The allocation of patients to robot-assisted or an open surgical approach may involve variables that influence postoperative infections resulting in a high risk of confounding bias. A low risk of bias was detected in the classification and deviations from interventions, as all four studies clearly defined the antibiotic prophylaxis schemes and surgical procedures.

The most influential risk of bias domain affecting the quality of evidence was the serious risk of bias due to missing data and bias in outcome measurement. None of the comparative studies reported about conducting a systematic follow-up evaluation at 30 days after surgery, thereby increasing the risk of bias due to missing data in outcome measurement [18].

We identified a heterogenous taxonomy for defining postoperative infectious complications that represents a serious bias in the reporting outcomes and follow-up results.

**Figure 2:**
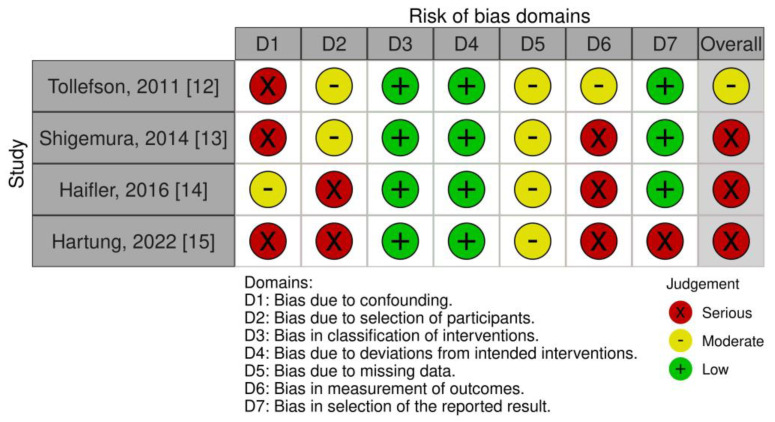
Risk of bias assessment for the four included non-randomized studies of interventions utilizing the ROBINS-I tool [10,12,13,14,15].

The risk of bias assessment for the included NRSI is visually depicted in the traffic-light plot (Figure 2) [10].

The JBI appraisal tool was used to assess the risk of bias in the included four case series (Table 3) [11]. All case series enrolled RALP patients on clear and consecutive criteria. However, apart from Ferrari et al., all studies were conducted retrospectively, potentially introducing bias in underreporting of outcomes and follow-up results [16]. Of note, Coelho et al.’s case series, including 2500 patients, consistently conducted a follow-up evaluation of patients at 6 weeks postoperatively, utilizing methods such as clinical examinations, phone calls, mail, or email [18]. This systematic follow-up approach may provide more reliable data on postoperative outcomes.

Additionally, none of the case series adhered to the infectious complication definitions outlined by the CDC, which increases the risk of underreporting outcomes and follow-up results. 

## 4. Discussion

### 4.1. Most Important Findings

This systematic review identified severe evidence gaps in the existing literature, which hinder the formulation of a definitive recommendation regarding AP in RALP. Our findings underscore that AP is being administered in RALP procedures without scientific evidence. The documented prevalence rates of infectious complications under the currently employed antibiotic regimens exhibit significant disparities, spanning from 0.6% to 6.6% [16,17]. The variation in prevalence rates is not attributed to varying AP protocols but is more likely due to limitations in the conduct of the studies and the inconsistent definitions of infectious complications.

### 4.2. Implications for Clinical Practice

Our findings align with previous studies on healthcare-associated infections (HAIs). In Europe (2011–2012), HAIs occurred at an average rate of 6% (CI: 5.7–6.3%) and in the United States, the rate was 4% (CI: 3.7–4.4%) [19,20]. The Global Prevalence Study on Infections in Urology (GPIU) found a healthcare-associated urinary tract infections (HAUTIs) prevalence of 9.4% in urology departments (2003–2010) [21]. These rates are, however, based on all types of urological surgeries with a wide spectrum of contamination categories. It is therefore a cause of concern that AP was administered in 87.3% of clean-contaminated surgeries like RALP, with second-generation cephalosporins most commonly used [4]. *Escherichia-coli* was the most frequent pathogen associated with HAUTIs, with a 33% resistance rate to cefuroxime globally [22]. Antimicrobial misuse contributes to microbial resistance [23] and our findings therefore highlight the urgent need for improved evidence related to AP in modern urologic surgery in general and to RALP in particular.

### 4.3. Understanding the Findings 

Caution is required when comparing infectious complication results in different studies. Retrospective analyses of patient records and non-uniform follow-up examinations may explain discrepancies. The prospective study by Ferrari et al. reported the highest infectious complications rates, with an overall rate of 6.6% and a UTI rate of 2.5% [16]. In contrast, the other seven retrospective studies reported considerably lower rates. This demonstrates the importance of underreporting bias in retrospective studies. We believe prospective assessment of patients for infectious complications and antibiotic side effects after surgical interventions yields more reliable results.

Infective complications can appear well beyond the inpatient stay. Among the included studies, only Coelho et al. conducted systematic post-discharge follow-up exams, while the remaining seven studies did not report on post-discharge follow-ups [18]. Too short follow-up periods may underestimate the occurrence of UTIs and other infectious complications.

Inconsistent definitions are critical to the rate of infectious complications. For example, the 9.4% HAUTI rate in the GPIU study encompassed cases of asymptomatic bacteriuria and those diagnosed solely based on clinical symptoms. Upon excluding these two categories, the HAUTI prevalence dropped to 5.1% [24]. Inconsistently applied definitions for infectious complications are a significant finding in our systematic review. As an example, Haifler et al. defined catheter-associated urinary tract infection (CAUTI) as symptomatic “cystitis or orchiepididymitis within 30 days following RALP”, whereas the official CDC classification considers a UTI as catheter-associated only if it occurs during catheter insertion or within 48 h after catheter removal [6,14]. Table 2 in our systematic review outlines the diverse definitions of infectious complications found in the reviewed literature.

### 4.4. Strengths and Weaknesses

The main strength of this study is the identification of RALP as a procedure where evidence related to AP is needed. We addressed the importance of definitions of UTI and the duration of the observation periods as well as patient demographics and surgical factors to identify risk factors for postoperative infections. In order to balance the benefits and harms of AP, we also studied the severity of infective complications and the rate of collateral effects of AP such as *C. difficile* infections. The fact that only a low number of studies was identified increases the importance of this work as a reference for future studies on antimicrobial prophylaxis in urological surgery. Due to identified limitations, a conclusive recommendation regarding the timing, dose duration and AP agent cannot be made.

### 4.5. Future Research

We need studies that provide high-level evidence on whether to use AP in RALP or not. We also need information about how to identify the right antibiotic in a given region or country and the dose and duration of the prophylaxis. Until now, guideline developers have focused on procedure-specific prophylaxis, but in the era of antimicrobial stewardship we must consider switching to a patient-specific tailored prophylaxis [25]. We therefore need more information about which patient characteristics increase the risk of infective complications. Finally, we need more information about risk factors for side effects like *C. difficile* infections and negative effects on the intestinal microbiome. This is necessary to balance benefits and harms and for patients to make wise choices. The information needs call for a sophisticated study design with a large number of patients where information is gathered continuously and embedded RCTs are designed as knowledge gaps are being filled. All studies must use generally accepted criteria for preoperative patient assessment such as urine culture and definitions of infection complications. 

## 5. Conclusions

This systematic review underscores the pressing requirement for more evidence about the role of AP in RALP. We need prospective studies that apply consistent and uniform criteria for measuring infectious complications and antibiotic-related side effects. Studies should be prospective with a systematic follow-up procedure adhering to the definitions of infectious complications set forth by the Centers for Disease Control and Prevention (CDCs). This will not only ensure reliability and validity of the data but also facilitate meaningful comparisons and a compilation of studies and provide better guidance for patients.

## Figures and Tables

**Table 1:** Results (AP, infectious complications): This table summarizes the applied antibiotic regimes, infectious complication rates und follow-up periods of all included studies.

1st Author, Year of Publication	Study Design/ Timespan	Population	Antibiotic Prophylaxis (AP)	Outcome Parameter	Outcome	Catheter Time, Days Mean ± SD	Remarks
Tollefson, 2011 [12]	retrospective cohort study (NRSI) (2004–2008)	1084 RALP vs. 4824 open PE	Short-term: Cephalexin within 1 h before surgical incision. AP was continued for 24 h after surgery.	SSI in RALP SSI in open PE UTI RALP Sepsis/ Bacteremia RALP *(within 30 days)*	6/1084 (0.6%) 216/4824 (4.5%) (*p* < 0.001) 17/1084 (1.6%) (*p* = 0.28) 1/1084 (0.1%) (*p* = 1.0)	not reported	SSI in RALP significantly less frequent and less severe compared to open PE
Shigemura 2013, Japan [13]	retrospective cohort study (NRSI) (2008–2012)	89 RALP (2010–2012) vs. 105 open PE (2008–2012)	Long-term: 3rd generation Cephalosporin or Ampicillin/Sulbactam in RALP. AP started 30 min prior to surgery and continued up to median 3 days.	SSI in RALP SSI in open PE *(within 30 days)*	1/89 (1.12%) 6/105 (4.77%) (*p* = 0.0876)	not reported	Group wanted to prove benefits of RALP regarding infectious complications as compared to open PE
Haifler 2016, USA/Israel [14]	retrospective cohort study (NRSI) (2010–2015)	229 RALP Prolonged AP: 60 RALP Single Shot SP: 169 RALP	Before 11/2011: Long-term AP: 2nd gen. Cephalosporin+ Aminoglycoside within 60 min. of incision followed by oral Fluoroquinolone until removal of catheter. After 11/2011: Single-dose AP: 2nd gen. Cephalosporin+ Aminoglycoside within 30 min. of incision.	CAUTI Prolonged AP Single Shot AP *(within 30 days)*	5/60 (8.3%) 15/169 (8.9%) (*p* = 0.89)	not reported	SS does not increase CAUTI rate compared to prolonged AP. “Within” 30–60 min of incision does not explain if the AP dose was administered pre- or intraoperatively.
Hartung 2022, Germany [15]	retrospective cohort study (NRSI) (2014–2015)	Total 376 Group 1: 216 (75% RALP and 25% open PE) Group 2: 160 (82.50% RALP and 17.50% open PE)	Long-term AP: Fluoroquinolone i.v. within 60 min. before incision, oral continued until removal of catheter. Single-dose AP: Ciprofloxacin or Cefuroxime within 60 min. before incision.	Postoperative wound infections and urinary tract infections per group *(within 30 days)*	Group 1: 21/216 (9.72%) Group 2: 19/160 (11.88%) (*p* = 0.5)	8.25 ± 6.44 8.25 ± 6.3 (*p* = 0.83)	Cohorts are mixed (RALP and open PE)
Ferrari 2020, Switzerland [16]	prospective case series (2011–2019)	317 RALP - 281/317 (88.6%) with PLND	Long-term AP: 3rd gen. i.v. Cephalosporin 30 min. before incision continued by oral Quinolone until postoperative day 7.	Total infectious complications Wound infections Lower urinary tract infection Respiratory tract infection Hyperpyrexia of unknown origin Infected Lymphocele Balanoposthitis *(within 90 days)*	*n* = 21/317 (6.6%) *n* = 2/317 (0.6%) *n* = 8/317 (2.5%) *n* = 2/317 (0.6%) *n* = 5/317 (1.6%) *n* = 3/317 (0.9%) *n* = 1 (0.3%)	6	“UTI” and “Genital/LUT infections”, not further specified
Ahmed 2012, USA [17]	retrospective case series (2004–2009)	1000 RALP	Short-term AP: Single preoperative i.v. dose followed by two postoperative doses (antibiotic agent not specified).	Total infectious complications Urinary tract infection *C. difficile* enterocolitis Upper respiratory infection *(within 30 days)*	6/1000 (0.6%) 2/1000 (0.2%) 3/1000 (0.3%) 1/1000 (0.1%)	7–8	Reports various complications in details but not about a single symptomatic lymphocele
Coelho 2010, Brazil [18]	retrospective case series (2002–2009)	2500 RALP	Single-dose AP: 1st gen. i.v. cephalosporin preoperatively.	Wound infection UTI after catheter removal acute epididymitis *(within 30 days)*	14/2500 (0.56%) 4/2500 (0.16%) 1/2500 (0.04%)	median 5	Complication rate might be underreported, only one very experienced surgeon
Hamada 2017, USA [19]	retrospective single arm study	865 RALPs + PLND (between 2008–2014)	Single-dose AP: 2 g cefazolin or 600 mg clindamycin (penicillin allergy) within 1 h of incision time.	Frequency of wound infection Infected lymphocele (LC) *(follow-up longer than 30 days: Median time to diagnosis was 6.8 ± 4.8 weeks)*	4/865 (0.46%) 26/865 (3%)	not reported	- Urinary tract infection not habitat for infected lymphocele. - Within 1 h of incision does not specify if AP dose was administered pre- or intraoperatively.

CAUTI = Catheter associated urinary tract infection, LUT = Lower urinary tract, NRSI = Non randomized study of intervention, PE = Prostatectomy, RALP = Robot assisted laparoscopic radical prostatectomy, SS = Single shot, SSI = Surgical cite infetion, UTI = Urinary tract infection.

**Table 2:** Infectious complications as quoted in the publications: This table presents the heterogeneity in the taxonomy of infectious complications across included studies.

1st Author, Year	Definition of Infectious Complication	Period of Infective Complication Rate	Author Comments
Tolleffson, 2011 [12]	Superficial and deep SSI: CDC criteria “Postoperative UTIs: Patients experiencing cystitis thought to be secondary to bacteriuria” “Sepsis or bacteremia”	within 30 days postoperatively	Use CDC criteria. Excluded patients without follow-up of at least 30 days.
Shigemura, 2013 [13]	Superficial, deep and organ/space SSI: CDC criteria Measurement of inflammatory laboratory parameters: WBC and CRP	within 30 days postoperatively	Use CDC criteria but do not report about a systematic follow-up after hospital discharge.
Haifler, 2016 [14]	“CAUTI: Symptomatic cystitis or orchiepididymitis within 30 days following RALP with or without positive urinary culture (i.e., over 10^5 CFU)”	within 30 days postoperatively	According to CDC a UTI is only catheter associated if the device is still in place or has been removed in the past 48 h.
Hartung, 2022 [15]	“UTI and wound infection” KISS* surveillance program [16] (German surveillance tool utilizing CDC definitions)	within 30 days postoperatively	Use CDC but did not apply them accordingly (no systematic follow-up carried out).
Ferrari, 2020 [16]	“UTI” “Wound infections” “Hyperpyrexia of unknown origin”, “Lymphocele infection” “Balanoposthitis”	up to 90 days postoperatively	Did not use CDC. Do not report about systematic follow-up at day 30.
Ahmed, 2012 [17]	“Infectious complications”, “UTI” “*C. difficile* enterocolitis” “Upper respiratory infection”	within 30 days postoperatively	Did not use CDC. Hospital records reviewed for complications within 30 days.
Coelho, 2010 [18]	“Wound infection” “UTI after catheter removal” “Acute epididymitis”	within 30 days postoperatively	Did not use CDC. Patients were contacted or examined 6 weeks postoperatively.
Hamada, 2017 [19]	“Infected Lymphocele” “Wound infection”	longer than 30 days	Only patients with symptomatic LC included.

CAUTI = catheter-associated urinary tract infection, CDC = centres for disease control and prevention, CRP = C-reactive protein, *KISS= Krankenhaus-Infektions-Surveillance-System, LC = lymphocele, RALP= Robot assisted laparoscopic radical prostatectomy, SSI = surgical site infection, UTI = urinary tract infection WBC = white blood cells.

**Table 3:** Risk of bias assessment for the four included case series using the JBI checklist.

JBI Checklist Questions (4)	Ferrari, 2021 [16]	Ahmed, 2012 [17]	Coelho, 2010 [18]	Hamada, 2017 [19]
Were there clear criteria for inclusion in the case series?	Yes	Yes	Yes	Yes
Was the condition measured in a standard, reliable way for all participants included in the case series?	Unclear	Unclear	Yes	Unclear
Were valid methods used for identification of the condition for all participants included in the case series?	Yes	Yes	Yes	Yes
Did the case series have consecutive inclusion of participants?	Yes	Yes	Yes	Unclear
Did the case series have complete inclusion of participants?	Yes	Yes	Yes	Unclear
Was there clear reporting of the demographics of the participants in the study?	Yes	Yes	Yes	Yes
Was there clear reporting of clinical information of the participants?	Yes	Yes	Yes	Yes
Were the outcomes or follow-up results of cases clearly reported?	No	No	No	Unclear
Was there clear reporting of the presenting site(s)/clinic(s) demographic information?	Yes	No	No	Yes
Was statistical analysis appropriate?	Yes	Yes	Yes	Yes

## Data Availability

Eva Falkensammer had full access to all the data in the study and takes responsibility for the integrity of the data and the accuracy of the data analysis.

## Supplementary Materials

The following supporting information can be downloaded at: https://www.mdpi.com/article/10.3390/antibiotics12121744/s1.

## Abbreviations

AP = Antimicrobial prophylaxis/ Antibiotic prophylaxis
AUA = American Association of Urology
CAUTI = Catheter-associated urinary tract infection
CDC = Centers for disease control and prevention
CINAHL = Cumulative Index to Nursing and Allied Health Literature
*Clostridioides difficile* = *C. difficile*
CRP = C-reactive protein
EAU = European Association of Urology
EMBASE = Excerpta Medica dataBASE
*Escherichia coli* = *E. coli*
GPIU = Global Prevalence Study on Infections in Urology
iLC = Infected lymphocele
JBI = Joanna Briggs InstituteKISS= Krankenhaus-Infektions-Surveillance-System
LC = Lymphocele
LUT = Lower urinary tract
MEDLINE = Medical Literature Analysis and Retrieval System Online
NRSI = Non-randomized study of intervention
RALP = Robot-assisted laparoscopic radical prostatectomy
RCT = Randomized controlled trial
ROBINS-I = Risk of Bias in Non-randomised Studies of Interventions
PC = Prostate cancer
PE = Prostatectomy
PLND = Pelvic lymph node dissection
PRIMSA = Preferred Reporting Items for Systematic Review and Meta-analysis
PROSPERO = International Prospective Register of Systematic Reviews
SSI = Surgical site infection
UTI = Urinary tract infection
WBC = White blood cells.
