# Supplementary material for: Antimicrobial Prophylaxis in Robot-Assisted Laparoscopic Radical Prostatectomy: A Systematic Review
Source: Antibiotics (Basel). 2023 Dec 16;12(12):1744. doi: 10.3390/antibiotics12121744 (PMC10740870; doi:10.3390/antibiotics12121744)
Supplement: Supplementary file 1 [file antibiotics-12-01744-s001.zip › antibiotics-2771601-supplementary.pdf]

Supplementary

Search strategies

Database: OVID Medline Epub Ahead of Print, In-Process & Other Non-Indexed Citations, Ovid MEDLINE(R) Daily and Ovid MEDLINE(R) 1946 to Present, EBM Reviews - Cochrane Central Register of Controlled Trials <December 2022>, EBM Reviews - Cochrane Database of Systematic Reviews <2005 to January 10, 2023>
Search Strategy:
----

1. exp prostatectomy/
2. prostatectom*.af.
3. ((remov* or resect* or surgical or surger* or operat* or RALP or RARP) adj3 prostat*).af.
4. or/1-3
5. Robotics/
6. robot*.af.
7. (remote* adj3 (surgical or surger* or operat*)).af.
8. or/5-7
9. 4 and 8
10. exp Anti-Infective Agents/
11. Antibiotic Prophylaxis/
12. exp Quinolones/
13. exp Carbapenems/
14. Disinfection/
15. exp Sulfonamides/
16. exp Aminoglycosides/
17. exp Iodophors/
18. (antibiotic* or antibacterial or anti bacterial or antiseptic* or antimicrobial* or anti infect* or antiinfect* or disinfect*).af.
19. (fluoroquinolone* or quinolone* or gentamicin* or amikacin or metronidazol* or flagyl).af.
20. (carbapenem* or trimethoprim* or sulphamethoxazol* or Co-amoxiclav).af.
21. (clavulanic acid or sulfonamid* or aminoglycosid* or cephalosporin* or Cefazolin* or Cefalexin* or Cefadroxil* or Cefaclor or Cefuroxim* or Cefoxitin* or Cefixim* or cefotaxim* or cefpodoxim* or ceftriaxon* or ceftazidim* or cefoperazon* or cefepim* or piperacillin* or norfloxacin*).af.
22. (ciprofloxacin* or ofloxacin* or tinidazol* or cephtriaxon* or ceftriaxon* or netilmicin* or netromycin*).af.
23. (cotrimoxazole or co-trimoxazole or sulfamethoxazole or beta lactam* or "β- lactam" or "β-lactam").af.
24. (povidone iodine or betadine or iodophor).af.
25. or/10-24
26. 9 and 25
27. remove duplicates from 26

****

Database: EMBASE

Embase <1974 to 2023 January 10>
Search Strategy:

----

1. exp prostatectomy/
2. prostatectom*.af.
3. ((remov* or resect* or surgical or surger* or operat* or RALP or RARP) adj3 prostat*).af.
4. or/1-3
5. Robotics/
6. robot*.af.
7. (remote* adj3 (surgical or surger* or operat*)).af.
8. or/5-7
9. 4 and 8
10. exp antiinfective agent/
11. antibiotic prophylaxis/
12. exp quinolone derivative/
13. tinidazole/
14. disinfection/
15. exp beta lactamase inhibitor/
16. (antibiotic* or antibacterial or anti bacterial or antiseptic* or antimicrobial* or anti infect* or antiinfect* or disinfect*).af.
17. (fluoroquinolone* or quinolone* or gentamicin* or amikacin or metronidazol* or flagyl).af.
18. (carbapenem* or trimethoprim* or sulphamethoxazol* or Co-amoxiclav).af.
19. (clavulanic acid or sulfonamid* or aminoglycosid* or cephalosporin* or Cefazolin* or Cefalexin* or Cefadroxil* or Cefaclor or Cefuroxim* or Cefoxitin* or Cefixim* or cefotaxim* or cefpodoxim* or ceftriaxon* or ceftazidim* or cefoperazon* or cefepim* or piperacillin* or norfloxacin*).af.
20. (ciprofloxacin* or ofloxacin* or tinidazol* or cephtriaxon* or ceftriaxon* or netilmicin* or netromycin*).af.
21. (cotrimoxazole or co-trimoxazole or sulfamethoxazole or beta lactam* or "β- lactam" or "β-lactam").af.
22. (povidone iodine or betadine or iodophor).af.
23. or/10-22
24. 9 and 23
25. case report/
26. case report.ti.
27. 25 or 26
28. 24 not 27

Database: CINAHL

1. (MH "Prostatectomy+")
2. TX prostatectom*
3. TX ((remov* or resect* or surgical or surger* or operat* or RALP or RARP) and prostat*)
4. S1 OR S2 OR S3
5. (MH "Robotics+")
6. TX robot*
7. TX (remote* and (surgical or surger* or operat*))
8. S5 OR S6 OR S7
9. S4 AND S8
10. (MH "Antiinfective Agents+")
11. (MH "Antibiotic Prophylaxis")

12. TX antibiotic* or antibacterial or anti bacterial or antiseptic* or antimicrobial* or anti infect* or antiinfect* or disinfect*
13. TX fluoroquinolone* or quinolone* or gentamicin* or amikacin or metronidazol* or flagyl
14. TX carbapenem* or trimethoprim* or sulphamethoxazol* or Co-amoxiclav
15. TX clavulanic acid or sulfonamid* or aminoglycosid* or cephalosporin* or Cefazolin* or Cefalexin* or Cefadroxil* or Cefaclor or Cefuroxim* or Cefoxitin* or Cefixim* or cefotaxim* or cefpodoxim* or ceftriaxon* or ceftazidim* or cefoperazon* or cefepim* or piperacillin* or norfloxacin*
16. TX ciprofloxacin* or ofloxacin* or tinidazol* or cephtriaxon* or ceftriaxon* or netilmicin* or netromycin*
17. TX cotrimoxazole or co-trimoxazole or sulfamethoxazole or beta lactam* or "β- lactam" or "β-lactam"
18. TX povidone iodine or betadine or iodophor
19. S10 OR S11 OR S12 OR S13 OR S14 OR S15 OR S16 OR S17 OR S18
20. S9 AND S19
21. S20 Limiters - Exclude MEDLINE records
